# Deciphering the Role of Shugoshin-Like Protein 1 in Lung Adenocarcinoma: A Comprehensive Analysis and *In Vitro* Study

**DOI:** 10.3389/fonc.2022.898920

**Published:** 2022-05-03

**Authors:** Yixiao Yuan, Juan Wang, Dahang Zhang, Lin Tang, Lincan Duan, Xiulin Jiang

**Affiliations:** ^1^Department of Thoracic Surgery, The Third Affiliated Hospital of Kunming Medical University, Kunming, China; ^2^Key Laboratory of Animal Models and Human Disease Mechanisms of Chinese Academy of Sciences & Yunnan Province, Kunming Institute of Zoology, Kunming, China

**Keywords:** shugoshin-like protein 1, lung adenocarcinoma, prognosis biomarker, cell proliferation, cell apoptosis

## Abstract

Shugoshin-like protein 1 (SGO1) has been characterized in its function in correct cell division and its role in centrosome cohesion in the nucleus. However, the underlying biological function and potential mechanisms of SGO1 driving the progression of lung adenocarcinoma remain unclear. In this study, we found that SGO1 was increased in LUAD tissues and cell lines. Upregulation of SGO1 expression was correlated with poor overall survival (OS), disease-free survival (DSS), and progression-free survival (PFS) in patients with LUAD. ROC curve analysis suggested that the AUC value of SGO1 was 0.983. Correlation analysis showed that SGO1 expression was related to immune infiltration in LUAD. Meanwhile, a potential ceRNA network was constructed to identify the lncRNA-MIR4435-2HG/miR-125a-5p/SGO1 regulatory axis in LUAD. Finally, we determine that SGO1 regulated the cell proliferation and cell apoptosis of lung adenocarcinoma *in vitro*. In conclusion, our data suggested that SGO1 could be a novel prognostic biomarker for lung adenocarcinoma.

## Introduction

Lung cancer is the leading cause of mortality among all malignancies worldwide. Among all types of lung cancer, lung adenocarcinoma (LUAD) is the most common subtype, accounting for 40%–50%, of lung cancer (1, 2). In the past several decades, considerable progress has been made in surgical treatment, radiotherapy, chemotherapy, molecular targeted therapy, and immunotherapy for lung cancer (3, 4).

However, most NSCLC patients are already in the advanced stage when it is initially diagnosed resulting in a short 5-year overall survival rate of patients (5). Therefore, elucidating the molecular mechanisms of lung oncogenesis and identifying new therapeutic targets or biomarker are essential for effectively preventing the development of lung cancer.

It has been confirmed that shugoshin 1 (SGO1) plays an important role in chromosome segregation maintaining centripetal adhesions during meiosis and mitosis (6). Previous studies have shown that SGO1 plays a crucial role in regulating the stability of microtubules (7). Several studies have shown that the depletion of SGO1 results in a premature dissociation of sister chromatids and mitotic arrest (8). It has been suggested that SGO1 was highly expressed in human prostate cancer tissues and cell lines. An increased SGO1 expression was correlated with preoperative prostate-specific antigen, lymph-node metastasis, advanced clinicopathological characteristics, and poor recurrence-free survival time (9). In our previous study, we developed a new method called CVAA (cross-value association analysis), which functions without a normalization and distribution assumption. We applied it to large-scale pan-cancer transcriptome data generated by The Cancer Genome Atlas (TCGA) project and successfully discovered numerous new differentially expressed genes (DEGs) (10). SGO1 is one of these DEGs. However, the underlying function and mechanisms of SGO1 in LUAD progression remain unclear.

No studies which evaluate the expression level, clinical significance, and prognostic and diagnostic values of SGO1 in LUAD can be retrieved currently. Therefore, the aim of this study was to determine the effect of SGO1 on the development of LUAD. In this study, we used The Cancer Genome Atlas (TCGA), the Genotype-Tissue Expression (GTEx), the Human Protein Atlas (HPA), and the Kaplan–Meier plotter web to determine SGO1 expression and its correlation with the prognosis. Furthermore, we used the single-sample gene set enrichment analysis (ssGSEA) method to analyze the correlation between SGO1 and immune infiltration. Besides, immunohistochemistry (IHC), qPCR, CCK8, colony formation, and flow cytometry assays were used to examine the biological function of SGO1 in LUAD progression.

## Materials and Methods

### TCGA Datasets

We downloaded the RNA expression data and related clinical contents from TCGA official website (https://portal.gdc.cancer.gov/) (11). We utilized this data analysis of the correlation between SGO1 expression and relevant clinical information, including pathological stage and TNM stage. Because the normal tissue sequencing data included in the TCGA are very limited and many patients lack transcriptome sequencing results for their normal tissues, we obtained data for normal tissues from the Genotype-Tissue Expression (GTEx) database. The above analyses were constructed using the R (v4.0.3) software package ggplot2 (v3.3.3). R software v4.0.3 and ggplot2 (v3.3.3) were used for visualization. R software v4.0.3 was used for statistical analysis.

### The Human Protein Atlas

The HPA (https://proteinatlas.org/) included various IHC images of protein of human gene information (12). In this finding, we explored the protein expression of SGO1 in lung cancer tissues.

### PrognoScan Database

PrognoScan (http://dna00.bio.kyutech.ac.jp/PrognoScan/index.html) is a new database for meta-analysis of the prognostic value of genes (13). In this study, we used PrognoScan to validation the prognostic values of SGO1 in lung cancer.

### LinkedOmics Database

LinkedOmics (http://www.linkedomics.org/login.php) is a publicly available portal that includes multi-omics data from all 32 TCGA cancer types and 10 Clinical Proteomics Tumor Analysis Consortium (CPTAC) cancer cohorts. In this study, LinkedOmics was employed to obtain the genes that were significantly positively correlated with SGO1 expression in TCGA-LAUD

### Kyoto Encyclopedia of Genes and Genomes and Gene Set Enrichment Analysis

Kyoto Encyclopedia of Genes and Genomes (KEGG) and gene set enrichment analysis (GSEA) were conducted to examine the biological and molecular functions of SGO1 across different cancer types using a total of 300 genes that were positively correlated with SGO1. All three analyses were performed using the R package Cluster Profiler. GSEA was also used to estimate the enrichment of various biological processes in each sample.

### Univariate and Multivariate Cox Regression Analyses and Kaplan–Meier Survival Analysis

Cox regression analysis, including univariate and multivariate analyses, was used to examine the prognostic value of SGO1 in LUAD. The univariate and multivariate Cox regression analyses were using the R package “forest plot” to exhibit the hazard ratio (HR), 95% CI, and P-value. The nomogram was constructed using the R package “rms.” In this research, the Kaplan–Meier method was utilized to examine the prognostic values of SGO1, miRNA, and lncRNA expression, employing R packages of survminer and survival.

### starBase Database

starBase v2.0 (http://starbase.sysu.edu.cn/) is a database which includes the RNA–RNA and protein–RNA interaction networks from 108 CLIP-seq data sets generated by 37 independent studies (14). In this study, starBase was used to predict the potential miRNAs and upstream lncRNAs of SGO1 and determine the correlation between miRNAs and SGO1 in LUAD. Furthermore, Pearson’s correlation analysis was used to determine the relationship between lncRNA and SGO1 expression in TCGA-LUAD.

### Cell Culture and Transfection

The BEAS-2B cell line was purchased from the Chinese Academy of Sciences Cell Bank (CASCB, Shanghai, China) and cultured in BEGM media (Lonza, Walkersville, MD, USA, CC-3170). Lung cancer cell lines, including SPC-A1, H358, A549, and H1299, were purchased from the Chinese Academy of Sciences Cell Bank (CASCB, China) with STR documents and were cultured in RPMI-1640 medium (Corning, Tewksbury, MA, USA) supplemented with 10% fetal bovine serum (FBS) and 1% penicillin/streptomycin. The siRNA for SGO1 and NC were transfected into cells. Transfection was performed using the Lipofectamine 3000 transfection reagent based on the manufacturer’s protocol. SGO1 siRNA: ACATGCACATACCTGAAAATG.

### Cell Proliferation Assay

Cell proliferation assay was performed as previously described (15). The indicated tumor cells were plated onto 12-well plates, and the cell numbers were subsequently counted each day using an automatic cell analyzer Countstar (Shanghai Ruiyu Biotech Co.).

### Real-Time RT-PCR Assay

The primer used in this study is as follows: β-actin-F: AAGTGTGACGTGGACATCCGC, β-actin-R: CCGGACTCGTCATACTCCTGCT, SGO1-F: AACTCAGCAGTCACCTCATCT, SGO1-R: TGCACCTACGTTTAGGCAGAG.

### Cell Flow Cytometry Assays

Annexin V FITC Apoptosis Detection Kit I (556547, BD, Guangzhou, China) was used to evaluate the cellular apoptosis according to the manufacturer’s instructions. For cell cycle analysis experiments, the indicated cells were digested and washed with PBS twice and then fixed in 75% alcohol overnight at −20°C. The fixed cells were washed three times and then stained with propidium iodide (PI) staining buffer at room temperature for 30 min in the dark, and then the cell cycle was analyzed using the FACSAria SORP machine (BD, USA).

### Immunohistochemical Staining

For immunohistochemical staining, the sections were deparaffinized in xylene and rehydrated through graded ethanol. Antigen retrieval was performed for 20 min at 95°C with sodium citrate buffer (pH 6.0). After quenching endogenous peroxidase activity with 3% H_2_O_2_ and blocking non-specific binding with 1% bovine serum albumin buffer, sections were incubated overnight at 4°C with the indicated primary antibodies. Following several washes, the sections were treated with an HRP-conjugated secondary antibody for 40 min at room temperature and stained with 3,3-diaminobenzidine tetrahydrochloride (DAB). SGOL1 antibody (Proteintech, Rabbit Polyclonal|: catalog number: 16977-1-AP, 1:200).

### Statistical Analyses

All statistical analyses were performed using R software, and ROC curves were used to detect SGO1 cutoff values using pROC packages. For data regarding the function of SGO1, GraphPad Prism 7.0 was used for statistical analyses.

## Results

### Expression Pattern of SGO1 in Human Cancers

**Figure 1:**
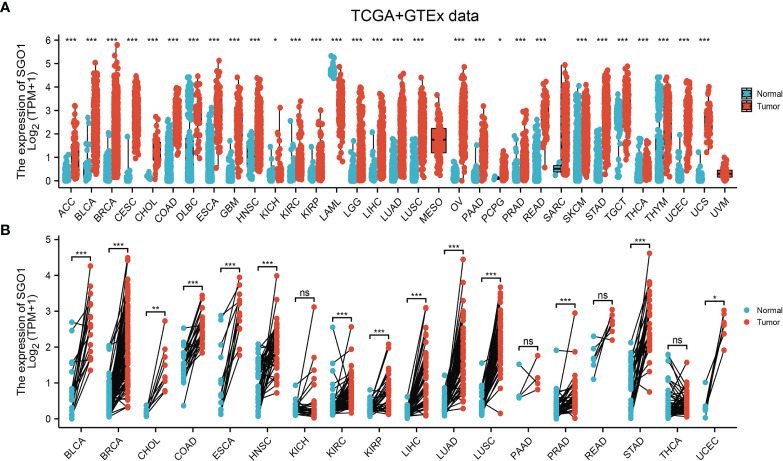
The expression pattern of SGO1 in pan-cancer. **(A, B)** The expression of SGO1 in pan-cancer examine by TCGA. NS: P > 0.05, ***P < 0.001. ACC: adrenocortical carcinoma, BLCA: bladder urothelial carcinoma, BRCA: breast invasive carcinoma, CESC: cervical squamous cell carcinoma and endocervical adenocarcinoma, CHOL: cholangiocarcinoma, COAD: colon adenocarcinoma, DLBC: lymphoid neoplasm diffuse large B-cell lymphoma, ESCA: esophageal carcinoma, GBM: glioblastoma multiforme, HNSC: head and neck squamous cell carcinoma, KICH: kidney: chromophobe, KIRC: kidney renal clear cell carcinoma, KIRP: kidney renal papillary cell carcinoma, LAML: acute myeloid leukemia, LUAD: brain lower-grade glioma, LIHC: liver hepatocellular carcinoma, LUAD: lung adenocarcinoma, LUSC: lung squamous cell carcinoma, MESO: mesothelioma, OV: ovarian serous cystadenocarcinoma, PAAD: pancreatic adenocarcinoma, PCPG: pheochromocytoma and paraganglioma, PRAD: prostate adenocarcinoma, READ: rectum adenocarcinoma, SARC: sarcoma, SKCM: skin cutaneous melanoma, STAD: stomach adenocarcinoma, TGCT: testicular germ cell tumors, THCA: thyroid carcinoma, THYM: thymoma, UCEC: uterine corpus endometrial carcinoma, UCS: uterine carcinosarcoma, UVM: uveal melanoma. NS: P > 0.05, *P < 0.05, **P < 0.01, ***P < 0.001.

To determine the mRNA expression pattern of SGO1 in various human cancers, we used TCGA and GTEx datasets to conduct analyses. Results suggested that SGO1 was upregulated in 26 of the 33 cancers compared with normal tissue **(** **Figure 1A****)**. We also estimated SGO1 expression in paired cancer tissues and adjacent normal tissues in pan-cancer based on TCGA datasets. Results confirmed that SGO1 expression was significantly higher in 14 of the 18 cancers compared with normal tissue **(** **Figure 1B****)**.

### SGO1 Was Overexpressed in Lung Adenocarcinoma

**Figure 2:**
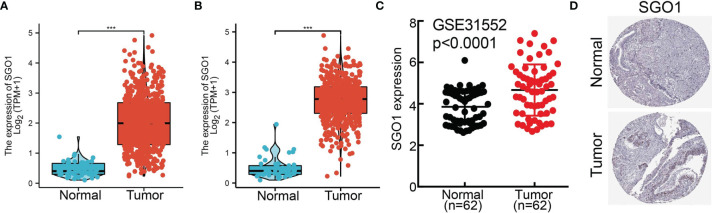
SGO1 was highly expressed in LUAD. **(A, B)** SGO1 was overexpressed in LUAD and LUSC examined by TCGA. **(C)** Relative SGO1 expression examined by the GEO dataset. **(D)** The protein level of SGO1 in lung cancer was determined by the HPA database. ***P < 0.001.

To examine the SGO1 expression level in LUAD, we analyzed SGO1 expression based on TCGA and HPA databases. We found that SGO1 was highly expressed in LUAD and LUSC **(** **Figures 2A, B****)**. Consistent with the above results, the GEO dataset also suggested that the SGO1 mRNA level was obviously increased in lung cancer tissues **(** **Figure 2C****)**. Furthermore, we showed that the SGO1 protein expression in LUAD was significantly increased in lung cancer tissues **(** **Figure 2D****)**.

### SGO1 Expression and Clinical Parameters of Lung Adenocarcinoma Patients

**Figure 3:**
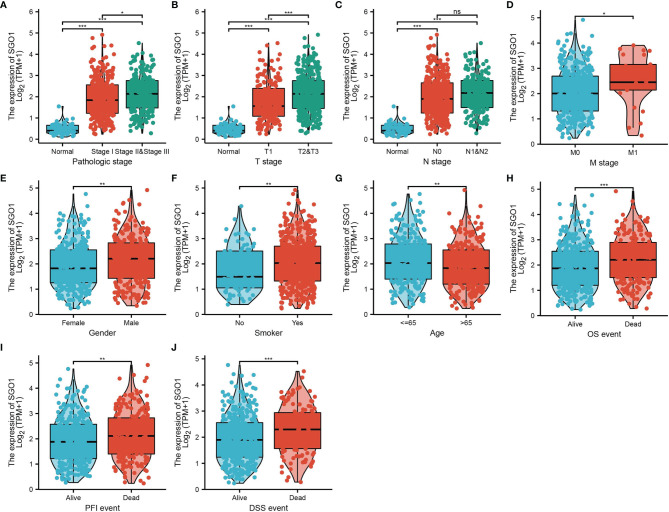
Clinical significance of SGO1 in LUAD. **(A–J)** Correlation between SGO1 expression and clinical parameters, including the pathological stage, TNM stage, smoking, age, gender, OS event, DSS event, and PFS event. NS: P <0.05, *P < 0.05, **P < 0.01, and ***P < 0.001.

The correlation between SGO1 expression and clinical parameters in LUAD samples was explored. As shown in **Table 1** and **Figures 3A–G**, SGO1 expression was significantly associated with pathological stage (P = 0.001), T stage (P < 0.001), N stage (P = 0.005), M stage (P = 0.009), gender (P = 0.001), residual tumor gender (P = 0.019), and age (P = 0.006). We also found that upregulation of SGO1 correlated with smoker, OS event, DSS event, and PFS event in LUAD **(** **Figures 3H–J****)**.

**Table 1:** Clinical characteristics of the LUAD patients.

Characteristic	Low expression of SGO1	High expression of SGO1	P
n	267	268	
T stage, n (%)			<0.001
T1	110 (20.7%)	65 (12.2%)	
T2	127 (23.9%)	162 (30.5%)	
T3	22 (4.1%)	27 (5.1%)	
T4	7 (1.3%)	12 (2.3%)	
N stage, n (%)			0.005
N0	187 (36%)	161 (31%)	
N1	45 (8.7%)	50 (9.6%)	
N2	25 (4.8%)	49 (9.4%)	
N3	0 (0%)	2 (0.4%)	
M stage, n (%)			0.009
M0	178 (46.1%)	183 (47.4%)	
M1	5 (1.3%)	20 (5.2%)	
Pathologic stage, n (%)			< 0.001
Stage I	166 (31.5%)	128 (24.3%)	
Stage II	59 (11.2%)	64 (12.1%)	
Stage III	31 (5.9%)	53 (10.1%)	
Stage IV	6 (1.1%)	20 (3.8%)	
Gender, n (%)			0.001
Female	162 (30.3%)	124 (23.2%)	
Male	105 (19.6%)	144 (26.9%)	
Primary therapy outcome, n (%)			0.106
PD	27 (6.1%)	44 (9.9%)	
SD	20 (4.5%)	17 (3.8%)	
PR	3 (0.7%)	3 (0.7%)	
CR	179 (40.1%)	153 (34.3%)	
Race, n (%)			0.936
Asian	3 (0.6%)	4 (0.9%)	
Black or African .American	28 (6%)	27 (5.8%)	
White	210 (44.9%)	196 (41.9%)	
Age, n (%)			0.028
≤65	116 (22.5%)	139 (26.9%)	
>65	145 (28.1%)	116 (22.5%)	
Residual tumor, n (%)			0.122
R0	178 (47.8%)	177 (47.6%)	
R1	5 (1.3%)	8 (2.2%)	
R2	0 (0%)	4 (1.1%)	
Smoker, n (%)			0.019
No	47 (9%)	28 (5.4%)	
Yes	211 (40.5%)	235 (45.1%)	
Age, median (IQR)	67 (60, 74)	64 (58, 71)	0.006

Logistic regression analysis also suggested that increased SGO1 expression was associated with T stage (T2 and T3 and T4 vs. T1) (P < 0.001), N stage (N1 and N2 and N3 vs. N0) (P = 0.006), M stage (M1 vs. M0) (P = 0.008), pathologic stage (stage III and stage IV vs. stage I and stage II) (P < 0.001), gender (male vs. female) (P <0.001), age (>65 vs. ≤65) (P = 0.022), and smoker (yes vs. no) (P = 0.015) **(** **Table 2****)**.

**Table 2:** Logistic regression analyzed the correlation between SGO1 expression and clinical pathological characteristics in LUAD.

Characteristics	Total (N)	Odds ratio (OR)	P value
T stage (T2 and T3 and T4 vs. T1)	532	2.180 (1.508–3.171)	<0.001
N stage (N1 and N2 and N3 vs. N0)	519	1.676 (1.159–2.434)	0.006
M stage (M1 vs. M0)	386	3.891 (1.537–11.893)	0.008
Pathologic stage (stage III and stage IV vs. stage I and stage II)	527	2.312 (1.498–3.619)	<0.001
Gender (male vs. female)	535	1.792 (1.272–2.530)	<0.001
Age (>65 vs. ≤65)	516	0.668 (0.471–0.944)	0.022
Smoker (yes vs. no)	521	1.869 (1.137–3.124)	0.015

### Analysis of the Diagnostic and Prognostic Values of SGO1 In LUAD

**Figure 4:**
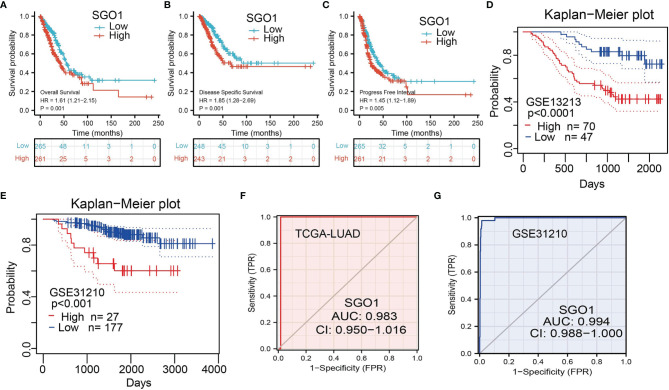
ROC and Kaplan–Meier curves of SGO1. **(A–C)** Kaplan–Meier survival curves showed that lung adenocarcinoma patients with high SGO1 expression exhibited poor overall survival, disease-specific survival, and progression-free survival of SGO1 in LUAD determine by TCGA-LUAD dataset. **(D–E)** Validation of the prognosis of SGO1 in LUAD using the GEO dataset. **(F,G)** ROC curves were used to determine the diagnostic value of SGO1 in lung adenocarcinoma.

The correlation between SGO1 expression and OS, DSS, and PFS in LUAD patients was examined by the Kaplan–Meier curve. We found that an elevated SGO1 expression was correlated with adverse OS, DSS, and PFS in patients with LUAD **(** **Figures 4A–C****)**. We also used the GEO dataset to validate the above results. We showed that upregulation of SGO1 expression was related to adverse clinical outcomes in patients with lung cancer **(** **Figures 4D, E****)**. We further explored the diagnostic significance of SGO1 in lung cancer, and ROC curve analysis was performed. ROC curve analysis confirmed that the AUC value of SGO1 is 0.983 **(** **Figure 4F****)**. The GEO dataset was also utilized to validate the diagnosis of SGO1 in lung cancer **(** **Figure 4G****)**. These results confirmed that SGO1 may be a promising biomarker for differentiating LUAD tissue from normal lung tissue.

### Validation of the Prognostic Value of SGO1 Based on Various Subgroups

**Figure 5:**
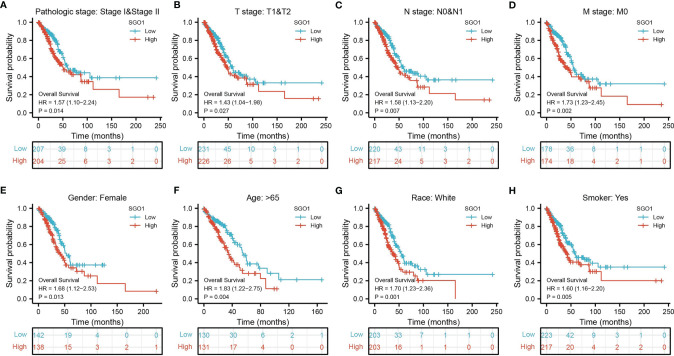
The overall survival of SGO1 based on diverse subgroups. **(A–H)** The overall survival of SGO1 based on diverse subgroups, including stage I and II, T1 and T2, N0 and N1, M0, gender, age >65, White, and smoker.

Prognostic values of the differential expression of SGO1 in diverse subgroups include pathological stage, TNM stage, gender, age, race, and smoker. Results confirmed that an increased SGO1 level is associated with poor prognosis **(** **Figures 5A–H****)**.

### Univariate and Multivariate Cox Regression Analyses of Different Parameters on Overall Survival

We performed univariate Cox regression analysis in TCGA-LAUD cohort to determine whether SGO1 expression level and pathologic stage might be valuable prognostic biomarkers. Univariate Cox regression analysis results show that higher expression of SGO1, pathologic stage, and TNM stage were associated with overall survival, disease-free survival, and progression-free survival in LUAD patients. To ascertain whether SGO1 expression level could be an independent prognostic factor for patients with LUAD, multivariate Cox regression analysis was performed. We confirmed that increased SGO1 expression was a significant independent prognostic factor in TCGA-LAUD cohort which directly correlated with adverse overall survival, disease-free survival, and progression-free survival **(** **Tables 3**-**5****)**.

**Table 3:** Univariate and multivariate Cox regression analyses of different parameters on overall survival in LUAD.

Characteristics	Total (N)	Univariate analysis		Multivariate analysis	
		Hazard ratio (95% CI)	P value	Hazard ratio (95% CI)	P value
Pathologic stage	518				
Stage I and stage II	411				
Stage III and stage IV	107	2.664 (1.960–3.621)	<0.001	5.960 (2.140–16.597)	<0.001
T stage	504				
T1	175				
T2 and T3	329	1.658 (1.175–2.341)	0.004	1.620 (1.029–2.553)	0.037
N stage	510				
N0 and N1	437				
N2 and N3	73	2.321 (1.631–3.303)	<0.001	0.452 (0.159–1.284)	0.136
M stage	377				
M0	352				
M1	25	2.136 (1.248–3.653)	0.006	0.347 (0.110–1.095)	0.071
Smoker	512				
No	72				
Yes	440	0.894 (0.592–1.348)	0.591		
Gender	526				
Female	280				
Male	246	1.070 (0.803–1.426)	0.642		
Age	516				
≤65	255				
>65	261	1.223 (0.916–1.635)	0.172		
SGO1	526	1.292 (1.120–1.490)	<0.001	1.200 (1.003–1.436)	0.047

**Table 4:** Univariate and multivariate Cox regression analyses of different parameters on disease-free survival in LUAD.

Characteristics	Total (N)	Univariate analysis		Multivariate analysis	
		Hazard ratio (95% CI)	P value	Hazard ratio (95% CI)	P value
Pathologic stage	483				
Stage I and stage II	389				
Stage III and stage IV	94	2.436 (1.645–3.605)	<0.001	2.235 (0.457–10.931)	0.321
T stage	473				
T1	168				
T2 and T3	305	1.815 (1.169–2.819)	0.008	1.659 (0.935–2.944)	0.083
N stage	475				
N0 and N1	410				
N2 and N3	65	1.971 (1.247–3.115)	0.004	0.980 (0.205–4.683)	0.980
M stage	344				
M0	323				
M1	21	2.455 (1.269–4.749)	0.008	1.044 (0.220–4.956)	0.957
Smoker	477				
No	69				
Yes	408	1.040 (0.602–1.796)	0.889		
Gender	491				
Female	262				
Male	229	0.989 (0.687–1.424)	0.954		
Age	481				
≤65	243				
>65	238	1.013 (0.701–1.464)	0.944		
SGO1	491	1.402 (1.169–1.683)	<0.001	1.300 (1.028–1.643)	0.028

**Table 5:** Univariate and multivariate Cox regression analyses of different parameters on progression-free survival in LUAD.

Characteristics	Total (N)	Univariate analysis		Multivariate analysis	
		Hazard ratio (95% CI)	P value	Hazard ratio (95% CI)	P value
Pathologic stage	518				
Stage I and stage II	411				
Stage III and stage IV	107	1.513 (1.105–2.071)	0.010	1.501 (1.070–2.106)	0.019
T stage	504				
T1	175				
T2 and T3	329	1.923 (1.407–2.629)	<0.001	1.751 (1.272–2.409)	<0.001
N stage	510				
N0 and N1	437				
N2 and N3	73	1.325 (0.914–1.919)	0.137		
M stage	377				
M0	352				
M1	25	1.513 (0.855–2.676)	0.155		
Smoker	512				
No	72				
Yes	440	0.968 (0.658–1.426)	0.870		
Gender	526				
Female	280				
Male	246	1.172 (0.901–1.526)	0.236		
Age	516				
≤65	255				
>65	261	1.023 (0.784–1.335)	0.867		
SGO1	526	1.276 (1.114–1.463)	<0.001	1.198 (1.035–1.388)	0.016

### KEGG Enrichment Analysis

**Figure 6:**
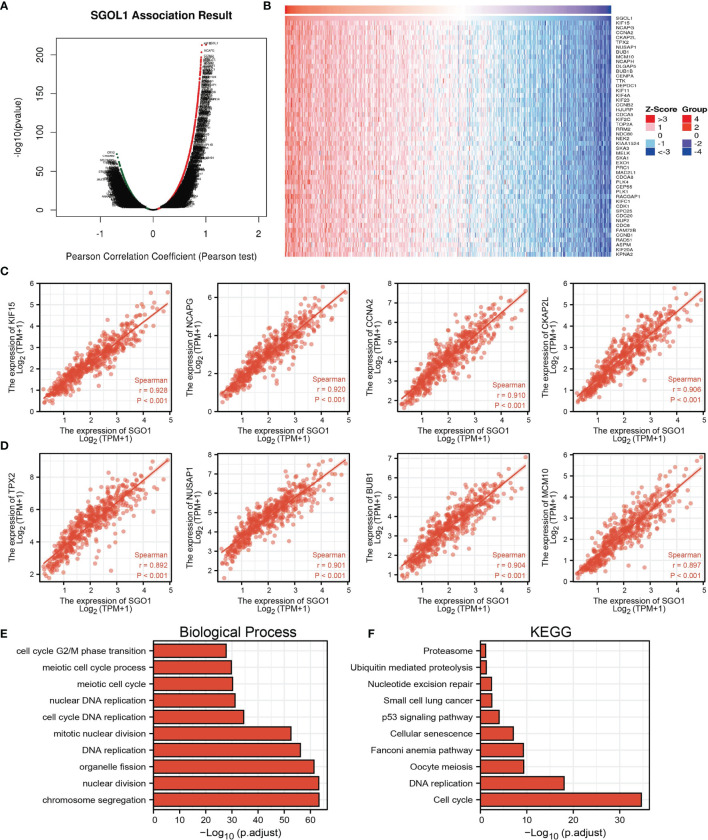
KEGG enrichment analysis of SGO1. **(A–D)** Genes that were significantly positively correlated with SGO1 expression in LUAD based on our TCGA-LAUD data. **(E, F)** GO and KEGG enrichment analysis of SGO1 in LUAD.

**Figure 7:**
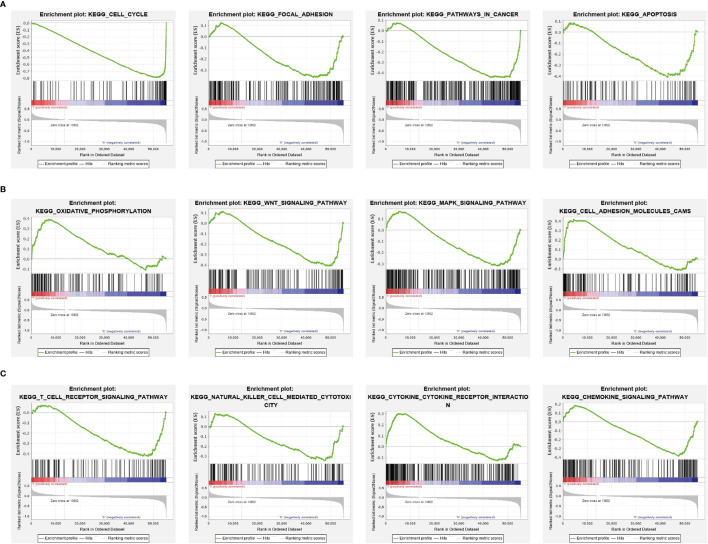
GSEA Identification of SGO1-related signaling pathways. **(A–C)** Identification of SGO1-related signaling pathways by GSEA software.

As shown in **Figures 6A, B**, the LinkedOmics database was utilized to obtain the top 100 genes that were significantly positively correlated with SGOL1 expression **(** **Figures 6A, B****)**. The correlation analysis of SGO1 expression and the top 8 co-expressed genes in TCGA LUAD is shown in **Figures 6C, D**. Notably, in terms of biological process, SGOL1 was enriched in cell-cycle G2/M phase transition, meiotic cell-cycle process, meiotic cell cycle, nuclear DNA replication, cell-cycle DNA replication, mitotic nuclear division, DNA replication, organelle fission, nuclear division, and chromosome segregation **(** **Figure 6E****)**. KEGG enrichment analysis suggested that these genes were involved in proteasome, ubiquitin-mediated proteolysis, nucleotide excision repair, non-small cell lung cancer, 53 signaling pathways, cellular senescence, Fanconi anemia pathway, oocyte meiosis, DNA replication, and cell cycle **(** **Figure 6F****)**. Gene set enrichment analysis (GSEA) also showed that pathways, including the cell cycle, focal adhesion, pathway in cancer, apoptosis, oxidative phosphorylation, Wnt signaling pathway, MAPK signaling pathway, cell adhesion molecules (cams), T-cell receptor signaling pathway, natural killer cell-mediated cytotoxicity, cytokine–cytokine receptor interaction, and chemokine signaling pathway were significantly enriched in the high SGO1 expression group **(** **Figures 7A–C****)**.

### Correlation Between SGO1 Expression and Immune Infiltration

**Figure 8:**
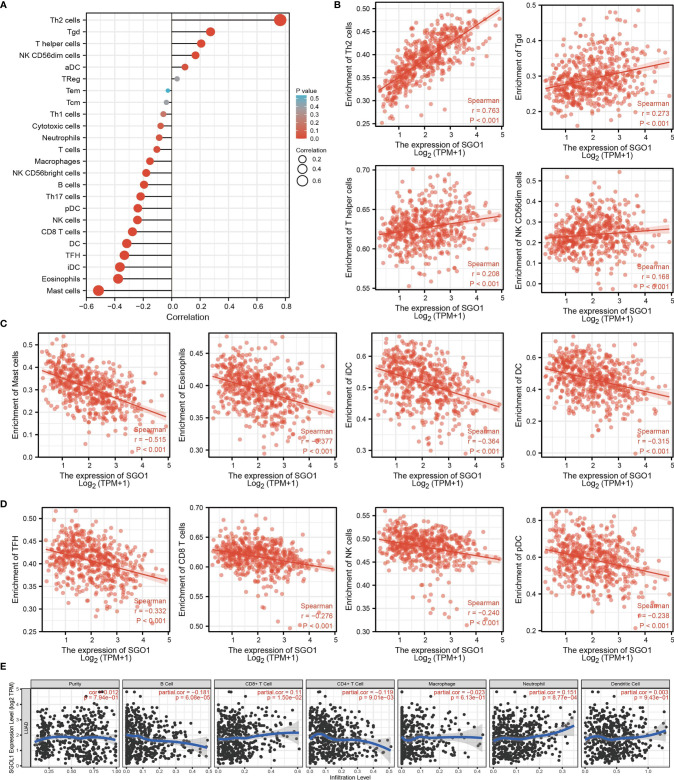
Correlation analysis of SGO1 expression and infiltration levels of immune cells in LUAD. **(A–D)** The correlation between SGO1 expression and the infiltration levels of 24 immune cells in LUAD. **(E)** Correlation of SGO1 expression with immune infiltration in LUAD.

Given that gene set enrichment analysis (GSEA) mentioned above showed that SGO1 may be correlated with immune response regulation, we therefore examined the association between SGO1 expression levels and immune cell infiltration. We found that SGO1 was positively associated with the infiltration of Th2 cells, Tgd, T helper cells, and NK CD56dim cells but negatively associated with the infiltration of pDC, NK cells, CD8 T cells, DC, TFH, iDC, eosinophils, and mast cells in LUAD **(** **Figures 8A–D****)**.

Our results also discovered that the high expression level of SGO1 was positively correlated with the infiltrating degree of CD8+ T cell and neutrophil and negatively correlated with the infiltrating degree of CD4+ T cell and B cell **(** **Figure 8E****)**.

### Construction of a Network of SGO1-Related ceRNA

**Figure 9:**
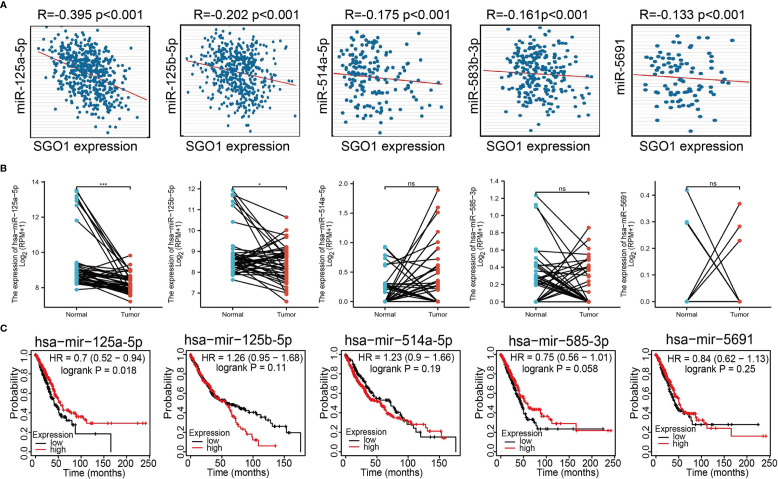
Analysis of the potential miRNAs of SGO1. **(A)** Correlations between SGO1 expression and miRNAs (miR-125a-5p, miR-125b-5p, miR-514a-5p, miR-585-3p, and miR-5691) in LUAD. **(B, C)** The expression level and prognosis of miRNAs (miR-125a-5p, miR-125b-5p, miR-514a-5p, miR-585-3p, and miR-5691) in LUAD .NS: P <0.05, *P < 0.05, and ***P < 0.001.

**Figure 10:**
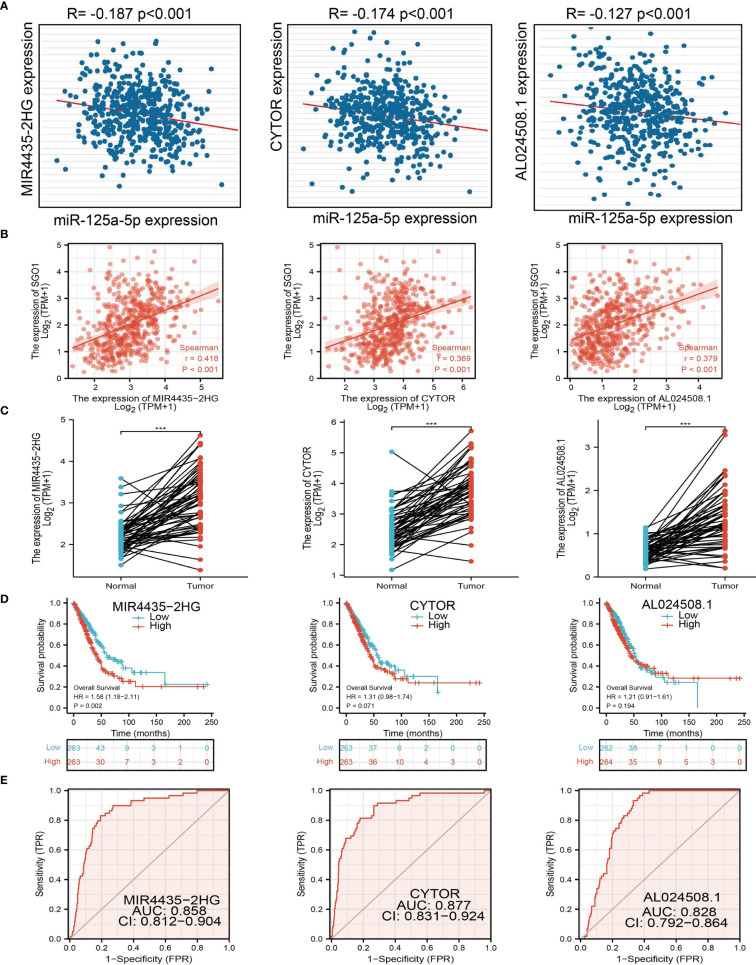
Analysis of the potential lncRNAs of miR-101-3p. **(A)** Correlations between miRNA-125a-5p expression and lncRNAs (MIR4435-2HG, CYTOR, and AL024508.1) in LUAD. **(B)** Correlations between SGO1 expression and lncRNAs (MIR4435-2HG, CYTOR, and AL024508.1) in LUAD. **(C–E)** The expression levels and prognostic and diagnostic values of lncRNAs (MIR4435-2HG, CYTOR, and AL024508.1) in LUAD ***P < 0.001.

The above results confirmed that SGO1 may participate in cancer progression in LUAD. To further determine the upstream potential molecular mechanism of SGO1 in LUAD, we therefore constructed an SGO1-related ceRNA network. We utilized the STRBase database to predict the potential miRNAs of SGO1 and identify 55 potential miRNAs **(** **Supplementary Table 1****)**; among these miRNAs, we showed that miR-125a-5p, miR-125b-5p, hsa-miR-514a-5p, hsa-miR-585-3p, and hsa-miR-5691 were significantly negative with the SGO1 expression in LUAD **(** **Figure 9A****)**. Further analysis revealed that only miR-125a-5p was downregulated in LUAD and that LUAD patients have a higher miR-125a-5p expression which was correlated with adverse clinical outcomes **(** **Figures 9B, C****)**. Therefore, we decided to select miR-125a-5p to conduct downstream analysis. We further examined the upstream lncRNA targets to construct the miRNA-125a-5p–lncRNA axis. Based on the ceRNA hypothesis, miRNAs have an opposite co-expression correlation with mRNAs and lncRNAs, whereas lncRNAs have a positive co-expression correlation with mRNA (16). Based on starBase and Pearson’s correlation analysis, we found that 3 three lncRNAs, including MIR4435-2HG, CYTOR, and AL024508.1, were negatively correlated with miR-125a-5p and positively correlated with SGO1 expression in LUAD, respectively **(** **Figures 10A, B****)**. We also found that MIR4435-2HG, CYTOR, and AL024508.1 were increased in LUAD, and a higher MIR4435-2HG expression was associated with poor prognosis in patients with LUAD **(** **Figures 10C, D****)**. ROC curve analysis confirmed that MIR4435-2HG, CYTOR, and AL024508.1 may be promising biomarkers for differentiating LUAD tissue from normal lung tissue **(** **Figure 10E****)**.

### Depletion of SGO1 Significantly Suppressed Proliferation of LUAD Cells

**Figure 11:**
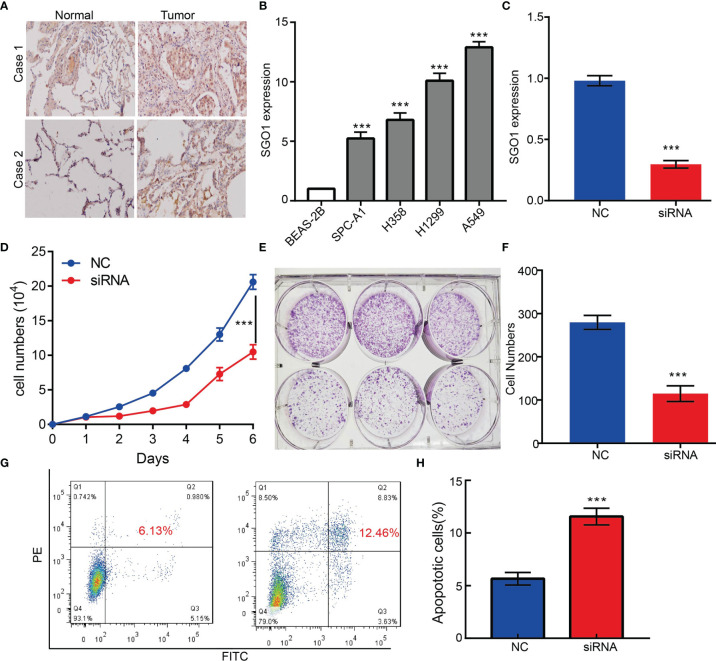
SGO1 modulates LUAD cell proliferation and migration *in vitro*. **(A)** The relative expression level of SGO1 in lung adenocarcinoma cancerous tissues examine by IHC assay. **(B)** The relative expression level of SGO1 in lung adenocarcinoma cancerous cell lines, including SPC-A1, H358, H1299, and A549 examined by real-time RT-PCR, compared to normal human bronchial epithelial cell line: BEAS-2B. **(C)** Establishment of SGO1 knockdown cell lines in A549 verified by real-time RT-PCR. **(D–F)** Knockdown of SGO1 significantly inhibits cell proliferation determined by CCK8 and colony formation assay. **(G,H)** Knockdown of SGO1 dramatically promotes cell apoptosis. ***P < 0.001. NC = negative control, siRNA = SGO1 siRNA. ***P < 0.001.

To examine the expression of SGO1, we detected SGO1 expression levels in LUAD tissues and cells lines using IHC and qRT-PCR assay. Results confirmed that SGO1 was significantly upregulated in lung cancer tissues and cell lines, especially in A549 cells (**Figures 11A, B****)**. To determine the biological function of SGO1 in lung cancer cells, small interfering RNAs (siRNAs) were used to specifically knock down SGO1 expression (**Figure 11C**). The growth curve and colony formation assays demonstrated that SGO1 depletion significantly inhibited the cell proliferation ability of LUAD (**Figures 11D–F**). Furthermore, to validate whether SGO1 is critical for cell apoptosis, we performed flow cytometry analysis and revealed that SGO1 knockdown led to increased apoptosis cells **(** **Figures 11G, H****)**. Collectively, these results demonstrate that SGO1 was highly expressed in LUAD and significantly affected their proliferation and cell apoptosis.

## Discussion

The centromere-related protein Shugoshin1 (SGO1) has been found to ensure the correct and orderly conduct of mitosis by protecting and maintaining centripetal adhesions during meiosis and mitosis (6). In this study, we analyzed the SGO1 expression, prognostic value, diagnostic values, and correlation with tumor immune cell infiltration in LUAD for the first time.

In this study, we found that SGO1 RNA and protein expressions were overexpressed in LUAD tissues. Increased SGO1 expression was correlated with pathological stage (P = 0.001) and TNM stage. Kaplan–Meier curves and univariate analysis confirmed that SGO1 expression is correlated with overall survival (OS), disease-free survival, and progression-free survival (PFS) in the LUAD patients of TCGA data. ROC curve analysis indicated that SGO1 may be a promising diagnostic biomarker for differentiating LUAD from normal tissues. Our findings are consistent with previous research. SGO1 was increased in human prostate cancer and correlated with the patients’ TNM stage, lymph node metastasis, distant metastasis, and poorer survival (17).

Logistic regression analysis also suggested that increased SGO1 expression was associated with T stage (T2 and T3 and T4 vs. T1) (P < 0.001), N stage (N1 and N2 and N3 vs. N0) (P = 0.006), M stage (M1 vs. M0) (P = 0.008), pathologic stage (stage III and stage IV vs. stage I and stage II) (P < 0.001), gender (male vs. female) (P < 0.001), age (>65 vs. ≤65) (P = 0.022), and smoker (yes vs. no). Next, the univariate and multivariate analysis results suggested that SGO1 expression was an independent prognostic biomarker for LUAD.

Previous studies reported that SGO1 promotes the proliferation and metastasis of prostate cancer *via* activating the AKT-mediated signaling pathway (17). It has been shown that SGOL1 variant B led to abnormal mitosis and resistance to taxane in NSCLC (18). In this study, we investigated the underlying mechanisms through which SGO1 affected the progression of LUAD. GSEA enrichment confirmed that SGO1 was significantly associated with cell cycle, focal adhesion, pathway in cancer, apoptosis, oxidative phosphorylation, Wnt signaling pathway, cytokine–cytokine receptor interaction, and chemokine signaling pathway.

It has been shown that SGO1 was highly expressed in prostate cancer and associated with adverse prognosis and immune infiltration (9). In this finding, we found that SGO1 expression was positively associated with the infiltration of Th2 cells, Tgd, T helper cells, and NK CD56dim cells but negatively associated with the infiltration of pDC, NK cells, CD8 T cells, DC, TFH, iDC, eosinophils, and mast cells in LUAD.

It has been well documented that ncRNAs, miRNAs, and lncRNAs are involved in the modulation of gene expression by communicating with each other through the ceRNA mechanism. To explore the upstream regulatory miRNAs of SGO1, we used starBase and found that has-miR-125a-5p may be an upstream miRNAs of SGO1. It has been confirmed that miR-125a-5p facilitates osteoclastogenesis *via* targeting TNFRSF1B (19). In gastric cancer, miR-125a-5p was found to promote gastric cancer cell growth and invasion by activating the Hippo pathway (20). We conducted the correlation analysis, expression analysis, and prognosis analysis. MiR-125a-5p was selected as the most potential upstream tumor-suppressive miRNA of SGO1. Finally, we also constructed the SGO1-related ceRNA network, which identified a lncRNA-MIR4435-2HG/miR-125a-5p/SGO1 regulatory axis. In fact, lncRNA MIR4435-2HG was reported to promote the migration and proliferation of NSCLS by increasing the TGF-β1 expression and activating β-catenin signaling, respectively (21, 22).

GSEA enrichment results show that SGO1 may play a central role in focal adhesion and cell apoptosis. We decide to examine the potential biological function of SGO1 in LUAD. *In vitro*, we found that SGO1 was upregulated in LUAD tissues and cell lines. Knockdown of SGO1 in A549 cells inhibited cell proliferation and increased apoptosis cells. Based on the above findings, we proposed that SGO1 exerts an essential function in regulating the pathologic progression of LUAD.

## Conclusion

This finding demonstrated, for the first time, the clinical significance and biological function of SGO1 in lung adenocarcinoma. In summary, SGO1 may serve as a promising diagnostic and prognostic biomarker for LUAD.

## Data Availability Statement

The original contributions presented in the study are included in the article/**Supplementary Material**. Further inquiries can be directed to the corresponding authors.

## Ethics Statement

This study was reviewed and approved by the Ethics Committee of The Third Affiliated Hospital of Kunming Medical University.

## Author Contributions

YY, JW, and DZ designed this work, and performed experiments, LT analyzed data. LD and XJ writed and revised the manuscript. All authors contributed to the article and approved the submitted version.

## Funding

This work was supported by the National Nature Science Foundation of China (82160508), Yunnan Applied Basic Research Projects (YNWRMY-2019-067, 2019FE001), and Yunnan Province Specialized Training Grant for High-Level Healthcare Professionals (D-201614).

## Conflict of Interest

The authors declare that the research was conducted in the absence of any commercial or financial relationships that could be construed as a potential conflict of interest.

## Publisher’s Note

All claims expressed in this article are solely those of the authors and do not necessarily represent those of their affiliated organizations, or those of the publisher, the editors and the reviewers. Any product that may be evaluated in this article, or claim that may be made by its manufacturer, is not guaranteed or endorsed by the publisher.
